# Prototyping ‘Typical Day’: Building a Gamified Experience To Reflect Immigrant Challenges

**DOI:** 10.1007/s10903-025-01711-z

**Published:** 2025-06-09

**Authors:** Diogo Martins, Maria Joana Campos, Marta Campos Ferreira, Carla Sílvia Fernandes

**Affiliations:** 1 Faculty of Engineering of the University of Porto, Portugal, Porto, Portugal; 2 Higher School of Nursing, Porto, Portugal, Porto, Portugal

**Keywords:** Empathy, Gaming, Immigrants, Participatory Games, Serious Play

## Abstract

This article describes the steps involved in creating a prototype with a gamified approach aimed at highlighting the challenges encountered by immigrants in foreign countries. This serious game sought to provide an interactive experience that mirrored the real-life obstacles faced by immigrants, fostering empathy among non-immigrant players in these scenarios, with the goal of improving attitudes toward immigrants. During the development phase of the game, a user-centered design approach was employed. The project was divided into several phases: understanding the context, comprehending user needs, iterative prototyping, and usability testing. Both immigrants and non-immigrants participated in the study, directly contributing to defining requirements and evaluating the game. The serious game “Typical Day,” designed to simulate everyday situations faced by immigrants through interactive scenarios and critical decisions, demonstrated positive acceptance in terms of usability and engagement. The results indicated that “Typical Day” provided an engaging and educational gaming experience, successfully balancing entertainment and information. Positive feedback from 45 non-immigrant participants highlighted its potential as an educational tool to raise awareness about the experiences of immigrants. However, further studies are needed to evaluate its long-term impact on attitudes and behaviors. In conclusion, this study contributes to the literature by addressing a gap in gamified approaches to immigrant challenges, laying the foundation for future developments in serious games aimed at promoting attitude change.

## Introduction

Mass immigration is a global phenomenon impacting nearly every country [1–3]. It is consistently ranked as a top concern among voters when assessing the primary challenges facing their nation or other political entities like the European Union, with more than a third of Europeans regarding it as the most critical issue for the EU [4]. This perspective has brought attitudes towards immigration, immigrants, and refugees to the forefront in many countries, particularly following the so-called ‘migration crisis’. In Western societies, these views are polarized: some recognize the benefits of immigration and view immigrants positively, while others regard these demographic shifts with suspicion and skepticism [1].

Public attitudes towards immigration have attracted significant academic interest and extensive empirical research in recent years [1, 3]. Attitudes refer to the feelings and dispositions of the population regarding a specific social issue, and results from previous research suggest that the most decisive indicator of attitudes towards immigrants is the sense of threat attributed to a particular immigrant community. The threats attributed to certain types of immigrants, or even to immigrants in general, can vary widely [5]. Understanding the negative and positive effects of attitudes towards migration is crucial, but it is even more important to actively seek effective strategies for transforming them [3].

Negative attitudes towards immigration are often the result of misinformation and a lack of education [6]. Research indicates that education plays a crucial role in moderating attitudes towards immigration, mitigating negative effects [7]. Education is therefore identified as a key individual factor in shaping attitudes towards migration, highlighting the need for educational policies that promote a deeper and more empathetic understanding of the challenges faced by immigrants [6, 7]. Education about immigration can teach and influence mindsets about the problems that immigrants face in a new society [6, 8, 9].

In the digital age, serious digital games have become a prominent tool for education, training, and learning. Serious games, designed with specific objectives, engage players in mastering new knowledge and skills by solving real-world problems through an interactive game [10–12]. In recent years, serious games and gamified resources designed to have an immediate and measurable impact on specific populations have emerged [2, 13–16]. Developers, designers, and researchers have made significant advancements with this new category of games, focusing on creating experiences that address real-world problems and drive tangible changes [13]. However, despite the prevalence of enhanced games discussed in this review study, as the authors note, none have fully captured the daily struggles of immigrants, particularly in the face of negative attitudes and other social barriers [6].

Building on this observation, several attempts at gamified approaches and serious games have been developed in recent years to address specific aspects of immigrant experiences. For example, “*Bury Me*,* My Love”* immerses players in the journey of a Syrian refugee, using a narrative based on real events to enhance understanding of the refugee reality [17]. Another game, “*Papers*,* Please”*, places players in the role of an immigration inspector, exposing them to ethical dilemmas related to border control and complex decisions about who is allowed to enter the country [18]. Additionally, games like “*Mind the Five”* [19] and “*Breathtaking Journey”* raise awareness about the vulnerabilities faced by immigrants [20]. These examples underscore the potential of gamification for addressing this issue, though greater awareness of the daily attitudes encountered by migrants is needed.

Although there are serious games such as *Bury Me*,* My Love* [17] and *Papers*,* Please* [18], which address specific refugee experiences and ethical dilemmas in migratory contexts, none explicitly portray the everyday situations of prejudice faced by immigrants in common social interactions. Thus, *Typical Day* stands out by specifically addressing these gaps, offering a unique experience that simulates and educates, about everyday negative attitudes.

Different attitudes towards immigrants are described, shaped by economic concerns about job competition, cultural threats to national identity, security fears, perceptions of increased crime, and competition for scarce resources [21]. However, to date, no games have been developed specifically aimed at changing these stereotypes about migrants [6]. Serious games can play a crucial role in education, transforming attitudes, fostering empathy, and shifting perspectives [6, 15].

Although significant advancements have been made in developing new resources that enable experiences addressing real-world problems and driving change, no digital resource has specifically addressed the immigrant experience, fostering actions of empathy and compassion. Aware of this gap and recognizing the potential of using a serious game in this context, the article outlines the steps to design and prototype *“Typical Day”.* This serious game aims to build a gamified experience that reflects the real-life challenges faced by immigrants, enhancing understanding and empathy among players in foreign countries.

## Methods

### Study Design

**Fig. 1:**
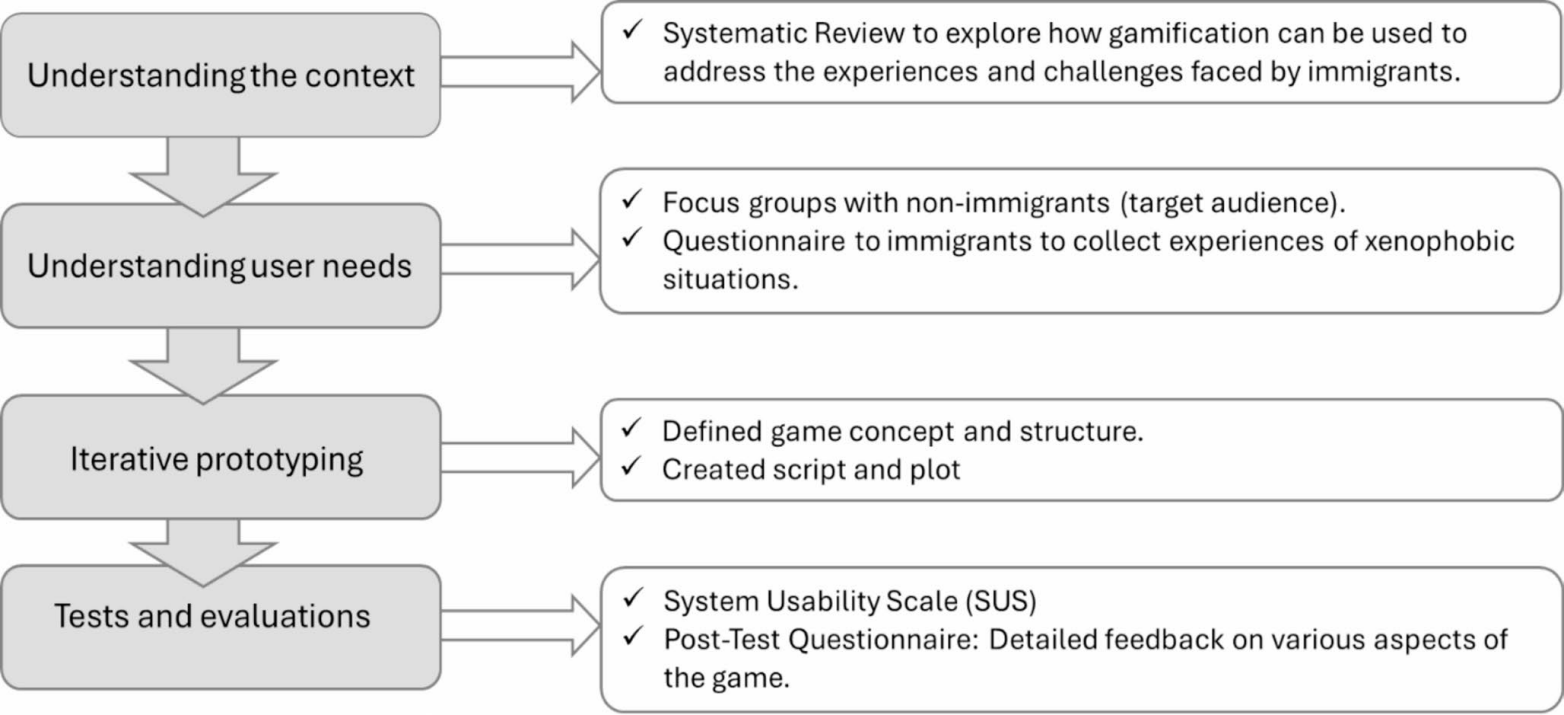
Study Design

The prototype was developed using a user-centered design methodology, integrating a set of four key tasks to achieve the proposed solution (Fig. 1). Gamification is increasingly recognized as a significant trend, with numerous theoretical frameworks advocating for user-centered design methods as fundamental. This approach is seen not just as a technique but as a comprehensive practice, philosophy, and discipline that deeply involves users in the design process [22]. The study was ethically approved, and participants were briefed on the study’s goals and objectives.

Anonymity and confidentiality of the data were assured. For the different stages of the study, immigrant participants were recruited through a Local Support Center for Migrant Integration and immigrant university students. Non-immigrant participants were recruited through social media and institutional mailing lists.

### Understanding the Context

To understand the context and ensure that the project development was aligned with the real issues it aimed to address, a systematic review study was conducted to analyze the use of gamified resources in exploring and addressing the experiences and challenges faced by immigrants. This phase was developed and published in a previous study [6].

### Understanding User Needs

Following the User-Centered Design methodology, it is essential to understand the users’ needs, as this step is crucial to ensuring that the game’s development reflects the users’ expectations and experiences. In this context, an integrated approach involving two main groups was undertaken: the non-immigrants, who make up the game’s target audience, and the immigrants, who contributed real experiences for the game’s content construction.

For the non-immigrants, two focus groups were organized. These were designed to gather their perceptions of immigrants, explore their views on using a game, and collect suggestions for the narratives.

Additionally, a questionnaire was administered to the immigrants, primarily aimed at collecting accounts of xenophobia experiences they had lived, which would be transformed into the game’s narratives. Moreover, this questionnaire sought to capture the participants’ opinions about the game in development, as well as recommendations for features and characteristics they considered relevant.

The collected data were analyzed in an integrated manner: the qualitative results, derived from the focus groups and open-ended questionnaire responses, were processed through content analysis, while the quantitative data from the questionnaire were explored using statistical methods. The qualitative analysis followed a thematic coding process, with two researchers independently conducting the initial coding, followed by consensus meetings to align the identified categories, thereby ensuring greater rigor and reliability of the results. This approach ensured alignment with the principles of User-Centered Design.

### Iterative Prototyping

Based on a literature review and interviews with immigrants and non-immigrants, key requirements for the game’s development were identified. This involved defining the game’s concept and structure, creating its script and plot based on findings from the previous phase, and developing its visual design. During this phase, it was possible to implement and code the computer game, using a game engine to integrate all aspects from the concept to the game logic defined in the previous stage, culminating in the first game prototype. The architecture of a serious game plays a crucial role in determining its functionality, performance, and user experience. The game included a narrative component with scenarios such as a run in the library, shopping at the grocery store, and volunteering at a charity. These scenarios were designed to engage players in immersive experiences that reflect real-life situations faced by immigrants, with the goal of fostering empathy and understanding among players.

### Tests and Evaluations

In the development of the game, conducting tests and validations is crucial to ensure its functionality, usability, and user satisfaction. This stage was carried out to allow for the evaluation of the game and how it was received by users in terms of overall experience.

The evaluation focused on the game’s three narratives: First Part (Library Run), the Second Part (Grocery Shopping), and the Third Part (Voluntary Work). These narratives were designed to simulate everyday situations faced by immigrants, aiming to promote awareness and empathy among players. Each part was individually assessed for its ability to provide an engaging and educational experience. Additionally, general aspects of the game, such as player engagement, enjoyment level, usability, challenge difficulty, and the balance between entertainment and educational content, were analyzed.

The evaluation tool was made available through an online form targeted at a sample of non-immigrants, the game’s primary audience. The post-test questionnaire included several sections, each designed to collect detailed feedback on different aspects of the game, allowing the identification of areas for improvement and enhancing its effectiveness as an educational tool.

The game’s usability was evaluated using an adaptation of the System Usability Scale (SUS), which includes 10 items providing a quick and general analysis of system usability. Moreover, specific items were added to investigate components of the game “A Typical Day,” such as players’ perceptions of increased awareness regarding immigration issues in Portugal and the emotional impact caused by the game.

Participants were given access to the game and the evaluation questionnaire via email, allowing them to experience the game at their own pace in a comfortable and familiar environment. This approach not only facilitated a more genuine interaction with the game but also likely resulted in more accurate and reflective feedback, as participants were able to engage with the game without the pressure of a controlled testing environment.

## Results

To facilitate the presentation of the results, the same steps presented in the methodology will be followed: Understanding the context, Understanding the needs, iterative prototyping, tests, and evaluations.

### Understanding the Context

The first phase, through a review study on the use of gamified resources in exploring and addressing the experiences and challenges faced by immigrants, revealed several important insights [6].The review study, published previously, revealed a moderate range of studies on gamification and the experiences of immigrants but did not identify any digital games that address the daily challenges faced by them due to barriers such as negative attitudes. This allowed us to move to the next stage of developing a serious game focused on exposing everyday prejudice against immigrants and exploring how gamification can foster empathy and support education against discrimination.

### Understanding User Needs

#### Focus Group

To investigate the perceptions of non-immigrants regarding the experiences of immigrants in Portugal and to explore the utility of an educational game, two focus groups were organized. The first group included 12 non-immigrant young adults, aged between 20 and 26, with varying levels of educational attainment. The second group consisted of 6 older participants, aged between 40 and 80, also non-immigrants, with different degrees of education, aiming to gain diverse perspectives on the topic.

The focus groups were conducted via Zoom, each lasting 60 min. The objective was to identify the participants’ perceptions of negative attitudes towards immigrants in Portugal, as well as to evaluate the perceived usefulness of a game as an educational tool. This included identifying the target audience, content, and ideas for the development of the game. The responses indicated that while young adults perceive xenophobia as a significant and growing problem in Portugal, primarily affecting immigrants from Latin American and African countries, the older participants believe the problem is less severe (Table 1). These contrasting perceptions underscore the complexity of attitudes towards immigration in Portugal and the potential of a game to educate and generate empathy among different age groups and social circles.

**Table 1:** Key findings from Non-Immigrant focus groups

Category	Focus Group 1 with Young Adults and Adults	Focus Group 2 with Old Adults
Perception of Negative Attitudes	- They believe that xenophobia is present in the daily lives of immigrants. - They highlight specific groups as particularly susceptible to prejudice, such as Latin Americans and Africans. - Some participants mentioned that they believe Eastern European immigrants do not face the same prejudice as other groups, being described as “hardworking people.” - Young adult participants’ perceptions of immigrant issues are mainly based on their own experiences or those of friends.	- They consider xenophobia to be almost nonexistent in the country. - However, they recognize the issue of underemployment among highly qualified immigrants. - Elderly participants’ perceptions of immigrant issues are mainly based on what they read in the news.
Perception of Serious Game Usage	- They see the game as a useful tool for educating and combating xenophobia.	- 11.1% believe that the serious game is unnecessary
Suggestions for the Game	- Include narratives and challenges that address the most common difficulties faced by immigrants. - Approximate duration of half an hour. - Include scalable challenges and scenarios of xenophobia. - A storytelling game with a narrative and a set of options for the user to choose from, which can influence the course of the story. - Utilize a reward system.	- The game should be accessible to everyone but primarily targeted at young people. - It should include challenges with dialogues between characters to convey the story.
Example of code units	- “I believe a game can help people understand what immigrants go through, as it puts us directly in their shoes.” (P5)	- “I never imagined that immigrants faced so many challenges to integrate.” (P3)

### Immigrants’ Experiences Survey Analysis

To address the issues and experiences of immigrants in Portugal, an online survey titled “Immigrant Experiences” was created and distributed to immigrants through a Local Center for Migrant Integration Support and to immigrant students at a university, totaling 29 responses. The characterization of the participants revealed that the majority are young adults (75.9%), predominantly from Brazil (37.9%) and from Mozambique and Ukraine (13.8% each). Most of the participants are students (65.5%) and have lived in Portugal for 5 years or more (24.1%) and came to Portugal alone (58.6%). (Table 2) The survey results showed that 62.1% of the participants experience some to a lot of xenophobia in Portugal, especially Brazilians and Mozambicans, as well as the unemployed, highlighting the need for a game that realistically addresses these issues.

As one participant stated: “When I was looking for an apartment, the landlord said he would not rent to me because of bad experiences with immigrants. This made me feel less valuable as a person.” (P4). Another participant shared: “I found it very difficult to integrate at first, not only because of the language barrier but also due to the attitudes I encountered on the streets and even in stores, where I was ignored or treated differently just for being a foreigner.” (P2). This reflects the daily discrimination, and the social and cultural barriers that immigrants often face and that the game aims to address.

The participants’ recommendations for the game’s content included exploring language barriers, cultural differences, the positive contributions of immigrants, the difficulties of being away from family, and the consequences of negative behaviors.

**Table 2:** Key results of immigrants’ experiences survey analysis

Category	Data
Participants	29
Age	- 18–30 years: 75.9% - 31–60 years: 20.7% - Over 60 years: 3.4%
Genders	- Male: 58.6% - Female: 41.4%
Countries of Origin	- Brazil: 37.9% - Mozambique: 13.8% - Ukraine: 13.8% - Others: 34.5%
Years of Residence in Portugal	- Less than 1 year: 20.7% - 5 years or more: 24.1%
Arrival in Portugal	- Alone: 58.6% - With family: 41.4%
Employment Status	- Students: 65.5% - Employed: 20.7% - Unemployed: 13.8%
Education Level	- Bachelor’s degree: 58.6% - Other levels: 41.4%
Experience with negative attitudes	- Feels a lot or some xenophobia: 62.1% - Feels little or no xenophobia: 37.9%
Opinions about Serious Game	- Useful: 66.7% - Not useful: 33.3% - Gameplay with narrative choices that impact outcomes - Increasing challenges based on the level of xenophobia faced by the characters - Rewards for integration and proper documentation.

With the data collected in the previous phase, it was possible to integrate both the experiences of immigrants and the opinions of non-immigrants on this topic. This approach ensured that the game is comprehensive and addresses the multifaceted nature of immigration, aiming to educate and foster empathy among players by exposing them to the various challenges and perspectives related to immigration in Portugal.

#### Iterative Prototyping

The development of a prototype is a time-consuming process that conditions the quality of the final product, making it crucial to define aspects to consider throughout the project. After thorough evaluation, the Godot engine was selected due to its free availability, ease of use, and suitability for developing mini games. Godot stands out for its ability to support both 2D and 3D development, offering the necessary tools for implementing a wide range of game functionalities.

**Fig. 2:**
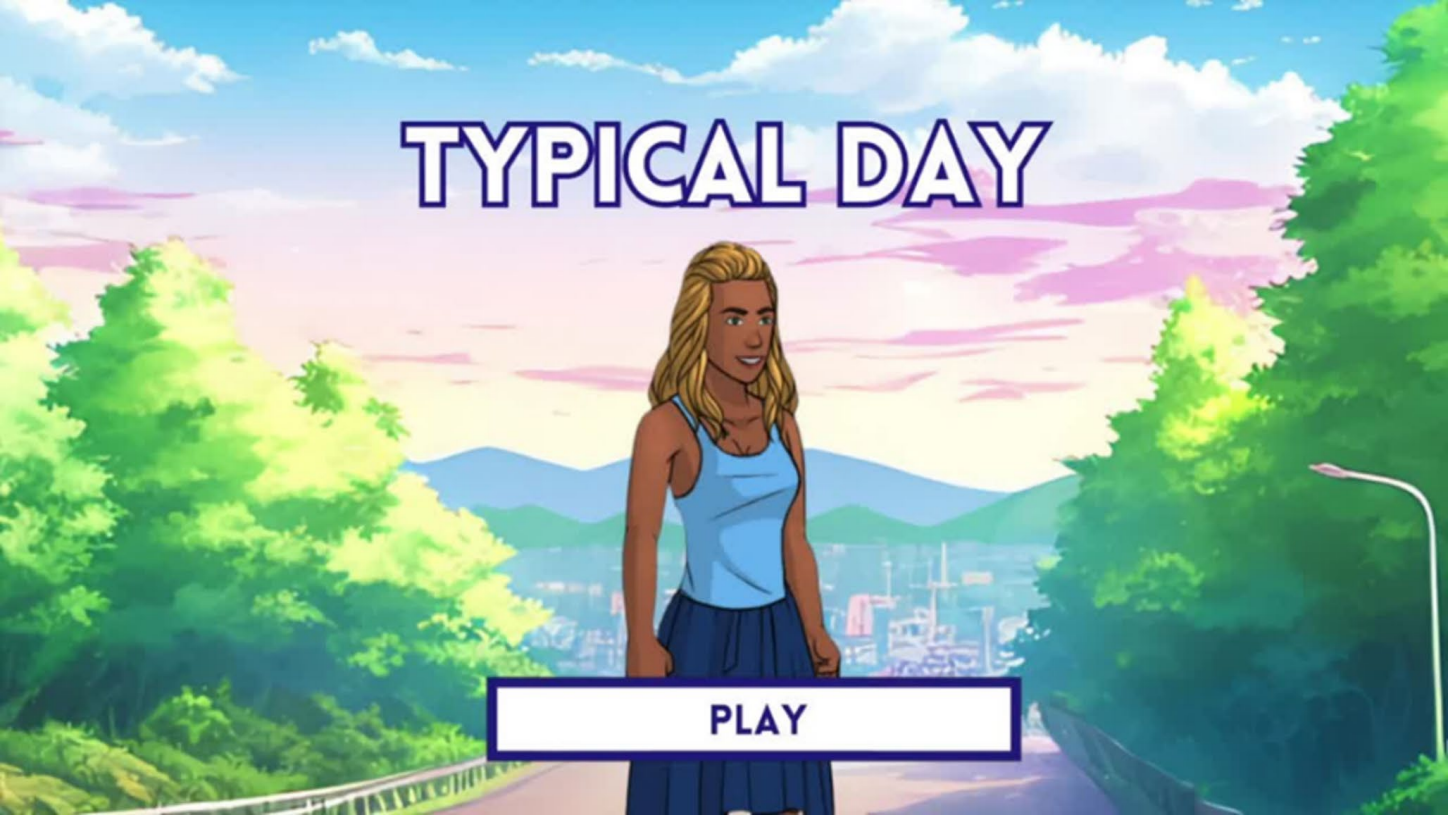
Game Screenshot

During the implementation phase, the Dialogic plugin was used to integrate branching and interactive dialogues, facilitating the creation of a cohesive and dynamic gameplay experience that aligns with the educational objectives of the project. Each phase of development was influenced by insights obtained in earlier stages, using continuous feedback to refine and improve each aspect of the game. The game was named “Typical Day” (Fig. 2).

**Fig. 3:**
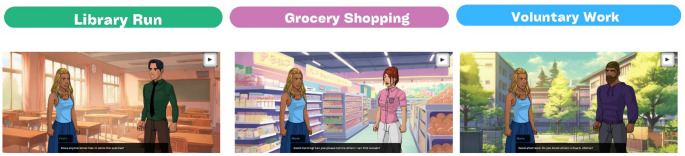
Narratives of the 3 Mini-Games

The game development process began with defining the overall structure of the game, which was divided into Narrative-based sections and Arcade and Minigames sections, interconnected by transitional segments. The game was titled “Typical Day.” Following this, the storyline and narrative scripts were crafted based on survey responses from immigrants and were subsequently validated by two of them upon completion. The mechanics for the arcade and minigames were then established, along with the logic for integrating these through the transitional sections. Content-wise, the game comprised three main parts: the Library Run Part, the Grocery Shopping Part, and the Voluntary Work Part (Fig. 3).

The game features three minigames that tackle different themes. In the “Library” minigame, Maria, a Brazilian student, interacts with her professor, Pedro, who displays negative attitudes. The setting is a classroom, and the objective of this Endless Runner-type minigame is for Maria to collect more than 20 reports before time runs out. This minigame simulates the pressure and the need to excel academically despite facing prejudice.

In the “Grocery Shopping” minigame, the game explores interactions in public services when the customer is an immigrant. Here, Maria faces Ana, a supermarket cashier who displays prejudice towards her. Set in a supermarket, this Platformer-type minigame challenges Maria to collect all the items on her shopping list before time runs out, while avoiding obstacles. This scenario illustrates the difficulties and unfair treatment immigrants may face in everyday tasks.

Lastly, in the “Voluntary Work” minigame, daily challenges faced by immigrants in public spaces are simulated. Maria encounters Francisco, a stranger who treats her with disdain and prejudice while she engages in volunteer work. This Chaser-type minigame takes place on the street, and Maria must collect 5 tickets before time runs out, avoiding capture by Francisco. This game represents the urgency and fear immigrants face when dealing with negative attitudes in their daily lives.

#### Tests and Evaluations

The evaluation of the game “Typical Day” was conducted using a post-test questionnaire and by employing an adaptation of the System Usability Scale (SUS). This questionnaire was divided into four subsections: one for each part of the game (Library Run, Grocery Shopping, and Voluntary Work) and a final subsection for the overall assessment of the game. Responses were given on a Likert scale of 1 to 5, where 1 represents “Strongly Disagree” and 5 represents “Strongly Agree” (Table 3).

The evaluation of the game “Typical Day” involved 45 non-immigrant participants. The gender distribution was 55.6% women, 42.2% men, and 2.2% identifying as other. The average age of the participants was 27.2 years. Most participants held a bachelor’s degree (57.8%), followed by 24.4% with a high school diploma, 15.6% with a master’s degree, and 2.2% with a doctorate.

Regarding familiarity with games, 35.6% of the participants reported feeling extremely familiar, 35.6% familiar, and 8.9% not familiar. Concerning comfort while playing, 31.1% felt completely comfortable, 35.6% comfortable, and 2.2% uncomfortable. As for issues related to immigrants, 4.4% of participants felt extremely familiar, 15.6% familiar, 24.4% somewhat unfamiliar, and 6.7% not familiar at all.

**Table 3:** Key results of test and evaluation

Evaluation	First Part	Second Part	Third Part	Overall Game
General Experience	4.44 (SD + 0.8)	4.47 (SD + 1)	4.69 (SD + 0.7)	4.54 (SD + 0.6)
Engagement	4.62 (SD + 0.2)	4.86 (SD + 0.4)	4.73 (SD + 0.9)	4.67 (SD + 0.2)
Enjoyment	4.31 (SD + 0.7)	4.38 (SD + 0.6)	4.89 (SD + 1)	4.47 (SD + 0.7)
Usability and Difficulty	4.00 (SD + 0.8)	3.91 (SD + 0.8)	4.42 (SD + 0.7)	4.34 (SD + 0.8)
Balance between Entertainment and Information	--	--	--	4.72 (SD + 0.2)
Integration and Consistency	--	--	--	4.56 (SD + 0.8)

The detailed results show that the overall experience of the game “Typical Day” was considered very enjoyable, with an overall average score of 4.54. The third part of the game stood out as the most enjoyable, achieving an average score of 4.69. Regarding engagement, the game received an average score of 4.67, indicating that the narrative parts were highly engaging, particularly the third part, which had an average score of 4.73. Fun was also rated highly, with an average score of 4.47, with the third part again being the most successful, scoring 4.89.

In terms of usability and difficulty, the average score was 4.34, suggesting that these aspects were adequate, with the third part being considered the most suitable (4.42). The balance between entertainment and information was rated with an average of 4.72, indicating a good balance between these elements. Finally, the integration and consistency of the game received an average score of 4.56, reflecting the participants’ perception that the game is well integrated and consistent.

Moreover, some participants in the study, through qualitative responses, highlighted that “the game made me reflect on situations that I previously would not have considered as xenophobia.” Another participant commented: “I was impressed by how the game managed to portray real-life scenarios in a way that made me feel personally involved. It made me realize how often we, as a society, overlook the subtle ways in which discrimination can occur, especially towards immigrants.” Some participants noted that, by simulating these situations, the game helped them understand that even small acts of resistance to inclusion—such as not offering help or showing discomfort during interactions—can constitute disguised forms of discrimination.

## Discussion

The study successfully achieved the goal of creating a prototype with a gamified approach, focused on raising awareness about the challenges faced by immigrants in foreign countries. This serious game aimed to provide an interactive experience that replicated the real challenges faced by immigrants, promoting understanding and empathy among non-migrant players in these contexts.

The results highlight how gamification elements can be effectively applied to promote social empathy. The “Typical Day” prototype stands out particularly for integrating real-life everyday situations, illustrating the potential of games to challenge stereotypes and foster empathy. Gamification thus emerges as a powerful tool to increase engagement [19] and facilitate understanding, allowing participants to interactively experience complex situations. This approach supports reflection on social issues and the development of empathy, especially in sensitive areas such as immigration [6, 17, 18].

Globally, an increasing number of people are on the move, making immigration one of the most pressing issues, often leading to substantial divisions in host societies [23]. European societies are experiencing a rising rate of immigration, and negative attitudes towards immigrants and immigration are influenced not only by national factors but also by individual factors, particularly education [7, 24]. This focus was central to the development of our prototype.

The development process included meeting all the scheduled requirements, ensuring that the prototype achieved the intended objectives and functionalities. With the development of this prototype, a foundation is established to address the issues faced by immigrants and to promote positive social emotions in players.

The development of this prototype went through several phases. The process of creating and designing a serious game should be systematic, organized, and participatory, constantly collecting feedback not only from the entire team but also from at least a sample of potential end users [22, 25].

In the first phase, a systematic review was conducted to understand what had been done in this field and what the results were [6]. After concluding that some games about immigrants were successful in achieving the goal of educating and raising awareness among players, and that, based on the material found, none of them addressed the daily problems of immigrants, the proposed solution was considered achievable and innovative in its specific theme.

Next, the opinions and interests of the target group of the game were gathered through two focus groups to understand the users’ needs. Information about the experiences and problems faced by immigrants in Portugal was also collected to inform the content to be conveyed. The results of both were used as a foundation for developing the game, alongside the study of the chosen game’s structure, gamification strategies, and game engine.

With all these foundations established, the game “Typical Day” was developed by defining its structure, creating prototypes for its parts, writing its scripts and storyline, designing its visuals, and implementing it in Godot. Regarding the gameplay experience, “Typical Day” proved to be engaging, enjoyable, usable, consistent, well-integrated, and well-balanced between entertainment and information. Serious games have been utilized in various contexts with serious purposes, especially in the healthcare field [19, 25] and other areas [26] and can be effectively applied to minimize stereotypes and inequalities faced by migrants [6]. Therefore, the proposed solution, in the form of a serious game, will be a catalyst for change that is intended to be applied in future phases through its large-scale implementation with pre- and post-facto studies.

### Limitations

For future work, it would be interesting to overcome some of the project’s limitations. Therefore, in terms of content, more issues faced by immigrants should be analyzed. After developing the game script based on these issues, it should be validated by a larger group to ensure that the game indeed reflects real situations that they can relate to.

Additionally, the game should undergo some modifications to become more engaging, applying feedback based on participants’ responses. Since the Volunteer Work part of the game was the most popular, the other two parts could have a similar structure and level of difficulty. The game’s visuals should also receive more attention, especially the Narrative-Based parts, with more character animations and facial expressions. The use of sound could also be considered to further engage players. Moreover, we recognize that the predominance of young participants with high educational attainment may represent a significant bias, potentially influencing the results positively. Future research should consider more demographically diverse samples, including different educational levels and age groups, to ensure greater applicability of the game across various sociocultural contexts.

Ultimately, the research could benefit from a larger sample size and a comparison with other educational tools or similar games to contextualize the effectiveness of the “A Typical Day” prototype relative to alternative approaches. Despite these limitations, the study represents a significant advance, laying solid foundations for future developments and more detailed long-term evaluations.

We suggest that future work should include longitudinal comparative studies using traditional educational tools to assess the specific effectiveness of the game in inducing attitude changes, combining quantitative evaluations with qualitative analyses.

## Conclusion

The article outlines the process of creating a prototype using a gamified approach, titled “Typical Day,” to reflect immigrant challenges. The development process followed a user center design, enabling continuous refinements based on user feedback. This approach allowed the team to quickly identify areas needing improvement and adjust the game as necessary to better meet players’ needs and expectations. Iterative prototyping was crucial for creating a game that was not only technically robust but also effective in conveying its message and achieving its educational and social goals.

Positive feedback from participants regarding usability and features demonstrated that the initial objectives of this research were met. However, assessing its long-term effectiveness and impact will require further investigation. This study addresses a gap in the literature and provides valuable insights for future developments.

Overall, the results indicate that the “Typical Day” game delivered an enjoyable and engaging gaming experience. The overall evaluation highlights that the game successfully achieved its usability goals and struck a balance between entertainment and education, raising awareness among players about the realities faced by immigrants. Future studies are expected to explore its potential for positively influencing attitudes.

## Data Availability

No datasets were generated or analysed during the current study.
